# Blockage of the lysosome-dependent autophagic pathway contributes to complement membrane attack complex-induced podocyte injury in idiopathic membranous nephropathy

**DOI:** 10.1038/s41598-017-07889-z

**Published:** 2017-08-17

**Authors:** Wei Jing Liu, Zhi-hang Li, Xiao-cui Chen, Xiao-lu Zhao, Zhen Zhong, Chen Yang, Hong-luan Wu, Ning An, Wei-yan Li, Hua-feng Liu

**Affiliations:** 1 Institute of Nephrology, and Zhanjiang Key Laboratory of Prevention and Management of Chronic Kidney Disease, Guangdong Medical University, Zhanjiang, Guangdong 524001 China; 2 Renal Research Institution of Beijing University of Chinese Medicine, and Key Laboratory of Chinese Internal Medicine of Ministry of Education and Beijing, Dongzhimen Hospital Affiliated to Beijing University of Chinese Medicine, Beijing, 100700 China

## Abstract

Dysregulation of autophagy-mediated podocyte homeostasis is proposed to play a role in idiopathic membranous nephropathy (IMN). In the present study, autophagic activity and lysosomal alterations were investigated in podocytes of IMN patients and in cultured podocytes exposed to sublytic terminal complement complex, C5b-9. C5b-9 upregulated the number of LC3 positive puncta and the expression of p62 in patient podocytes and in C5b-9 injuried podocyte model. The lysosomal turnover of LC3-II was not influenced, although the *BECN1* expression level was upregulated after exposure of podocytes to C5b-9. C5b-9 also caused a significant increase in the number of autophagosomes but not autolysosomes, suggesting that C5b-9 impairs the lysosomal degration of autophagosomes. Moreover, C5b-9 exacerbated the apoptosis of podocytes, which could be mimicked by chloroquine treatment, indicating that C5b-9 triggered podocyte injury, at least partially through inhibiting autophagy. Subsequent studies revealed that C5b-9 triggered lysosomal membrane permeabilization, which likely caused the decrease in enzymatic activity, defective acidification of lysosomes, and suppression of DQ-ovalbumin degradation. Taken together, our results suggest that the lysosomal-dependent autophagic pathway is blocked by C5b-9, which may play a key role in podocyte injury during the development of IMN.

## Introduction

During the past decade, there has been a remarkable rise in the frequency of idiopathic membranous nephropathy (IMN), which might ultimately predominate over IgA nephropathy to become the leading type of primary glomerulopathy in the near future in some developing countries, including China with a 13% annual increase^1^. However, the prognosis of IMN still falls far short of physician’s expectations. Many patients remain refractory for proteinuria and nephrotic edema throughout the disease course, and may progress to uremia in the final stage^2^. Recent landmark studies have shown that M-type phospholipase A2 receptor (PLA2R) is exposed at the podocyte’s surface to induce the production of autoantibodies under the influence of certain factors (e.g., viruses and pollutants), and then the complement system is activated by the subepithelial immune complex under the PLA2R antigen–antibody reaction^3, 4^. Complement activation leads to assembly of the membrane attack complex C5b-9 on podocytes, which is essential for sublethal damage to the intrinsic renal cells^5^. Several studies have reported that sublethal C5b-9 does not simply disrupt the plasma membrane of podocytes but also triggers a variety of biological responses, including the release of pro-inflammatory mediators, activation of Ca^2+^ influx-dependent protein kinases (such as protein kinase C, mitogen-activated protein kinases, extracellular signal-regulated kinase, and c-Jun N-terminal kinase), and over-production of reactive oxygen species (ROS) inside the cell^6–9^. Despite years of research, the specific pathophysiological mechanisms underlying C5b-9-induced podocyte injury have not yet been elucidated. Thus, more research is needed to uncover the pathogenesis or cellular processes underlying IMN toward the development of new treatment modalities and strategies for this devastating disease.

Autophagy, an evolutionarily conserved, lysosomal-mediated mechanism of intracellular degradation, is responsible for degrading intracellular protein aggregates and damaged organelles. Disruptions in normal autophagy that lead to autophagic stress may result in cellular injury and even cellular death^10^. Podocytes exhibit a high level of basic autophagy under physiological conditions relative to the basal level of autophagy existing in other intrinsic renal cells^11^. This situation suggests that the intracellular degradation system may be extremely important when podocytes suffer from sublethal damage caused by the membrane attack complex. Indeed, it has been suggested that autophagic activation plays a role in reducing podocyte injury and can even delay the progression of some podocyte injury-associated renal diseases^12, 13^. However, the changes in the autophagy process occurring in podocytes under the state of IMN remain unclear.

In the present study, an alteration of autophagic pathway was evaluated in renal tissues from patients with IMN. In addition, the activity and action of autophagy were studied in a sublytic C5b-9 membrane attack model after exposure of podocytes to the zymosan-treated serum, since zymosan could promote rapid C3 cleavage through assembly and protection of the amplification convertase on its surface and produce sera enriched with soluble C5b-9^14–16^. These results might provide a basis for further understanding the mechanism of podocyte damage in the pathogenesis of IMN.

## Results

### Autophagic vacuoles are accumulated in podocytes of patients with IMN

The clinical characteristics of 17 IMN patients and 14 control patients are presented in Table 1. The 24-h urinary protein excretion, serum triglyceride, and total cholesterol levels were significantly increased, while the serum albumin level was decreased in patients with IMN. There was no statistical significance in hemoglobin, serum creatinine, blood urea nitrogen, and serum uric acid levels between the IMN group and the control group.

**Table 1:** Clinical characteristics of the enrolled patients.

Groups	Control	Idiopathic membranous nephropathy	P-value
Sample size	14	17	—
Male sex (%)	8.0 (57.1%)	11.0 (64.7%)	0.74
Age (years)	34.6 ± 13.6	41.6 ± 20.3	0.26
Hemoglobin (g/L)	127.7 ± 21.3	127.2 ± 21.2	0.95
Total cholesterol (mmol/L)	4.2 ± 1.5	10.0 ± 3.5	<0.001
Serum albumin (g/L)	39.9 ± 4.3	20.9 ± 7.9	<0.001
Blood urea nitrogen (mmol/L)	4.7 ± 1.3	6.3 ± 3.4	0.09
Serum uric acid (μmol/L)	356.7 ± 151.9	367.9 ± 100.8	0.82
Triglyceride (mmol/L)	1.2 (0.7–2.3)	2.1 (1.4–3.6)	<0.05
Serum creatinine (μmol/L)	72.0 (52.8–85.0)	73.0 (44.5–105.0)	0.78
24-h urinary protein (g)	0.1 (0.05–0.3)	3.8 (1.5–4.8)	<0.001

**Figure 1:**
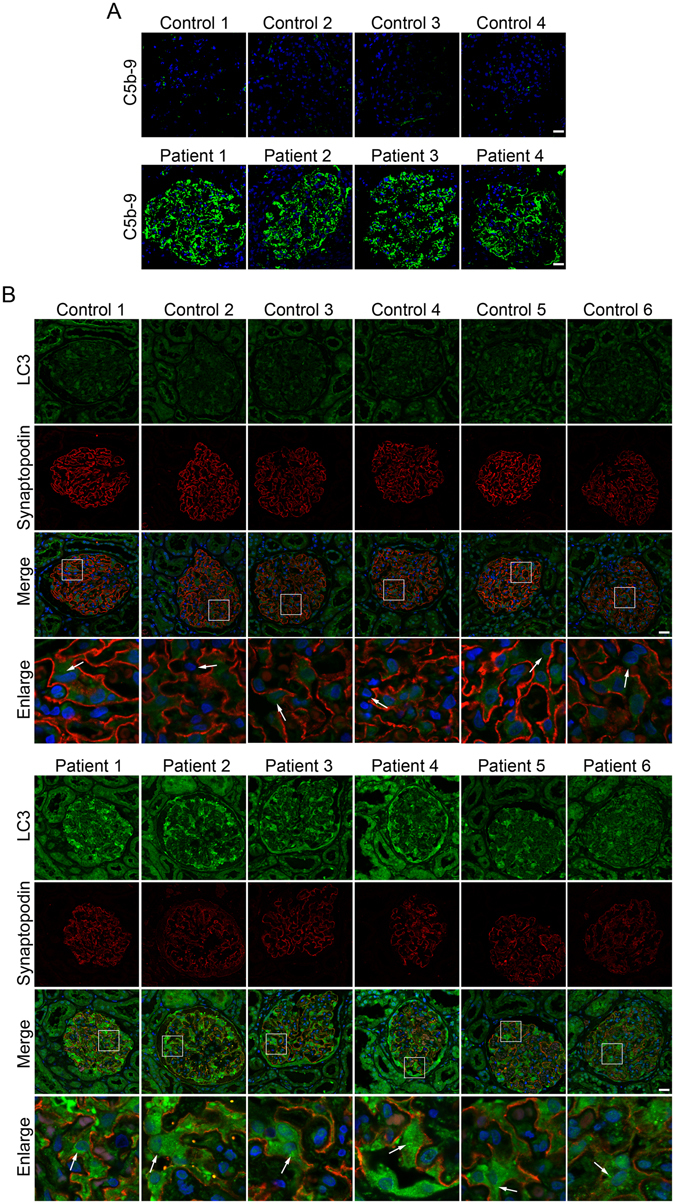
Autophagic vacuoles accumulate in podocytes of patients with idiopathic membranous nephropathy (IMN). (**A**) Immunofluorescent staining showing the deposition of linear C5b-9 in the glomerular basement membrane in patients with IMN compared with controls. (**B**) Immunofluorescent analysis of LC3-positive puncta (green) accumulated in podocytes of patients with IMN compared with controls. The podocytes in glomeruli were identified by immunofluorescent double-labeling with synaptopodin (red), a podocyte foot process-specific protein. The nuclei were stained with DAPI (blue). Images were obtained using a confocal microscope. The glomeruli presented in (**A**) may not always be the same as those shown in (**B**). White arrows indicate the expression of LC3-positive puncta in podocytes. Scale bar, 25 μm.

Before autophagosome detection, C5b-9 deposition was first assessed in all 31 renal tissues. We found massive deposition of the C5b-9 complement component in all renal tissues from patients with IMN, whereas no C5b-9 deposition was detected in the control renal tissues (Fig. 1A). Next, the expression of microtubule-associated protein 1 light chain 3 (LC3), a key marker protein recruited into the autophagosome membranes during induction of autophagy, was assessed in podocytes of renal specimens by immunofluorescent staining. We found that more LC3-positive vacuoles were accumulated in the podocytes of renal tissues from patients with IMN compared with those from controls (Fig. 1B). However, the strong background fluorescence was also observed in renal tissues from patients with IMN, which is likely the unspecific staining. To identify podocytes in the glomeruli, the samples were labeled with synaptopodin, a podocyte foot process-specific protein^17^, by double immunofluorescent staining.

### Autophagy is inhibited in podocytes of patients with IMN

**Figure 2:**
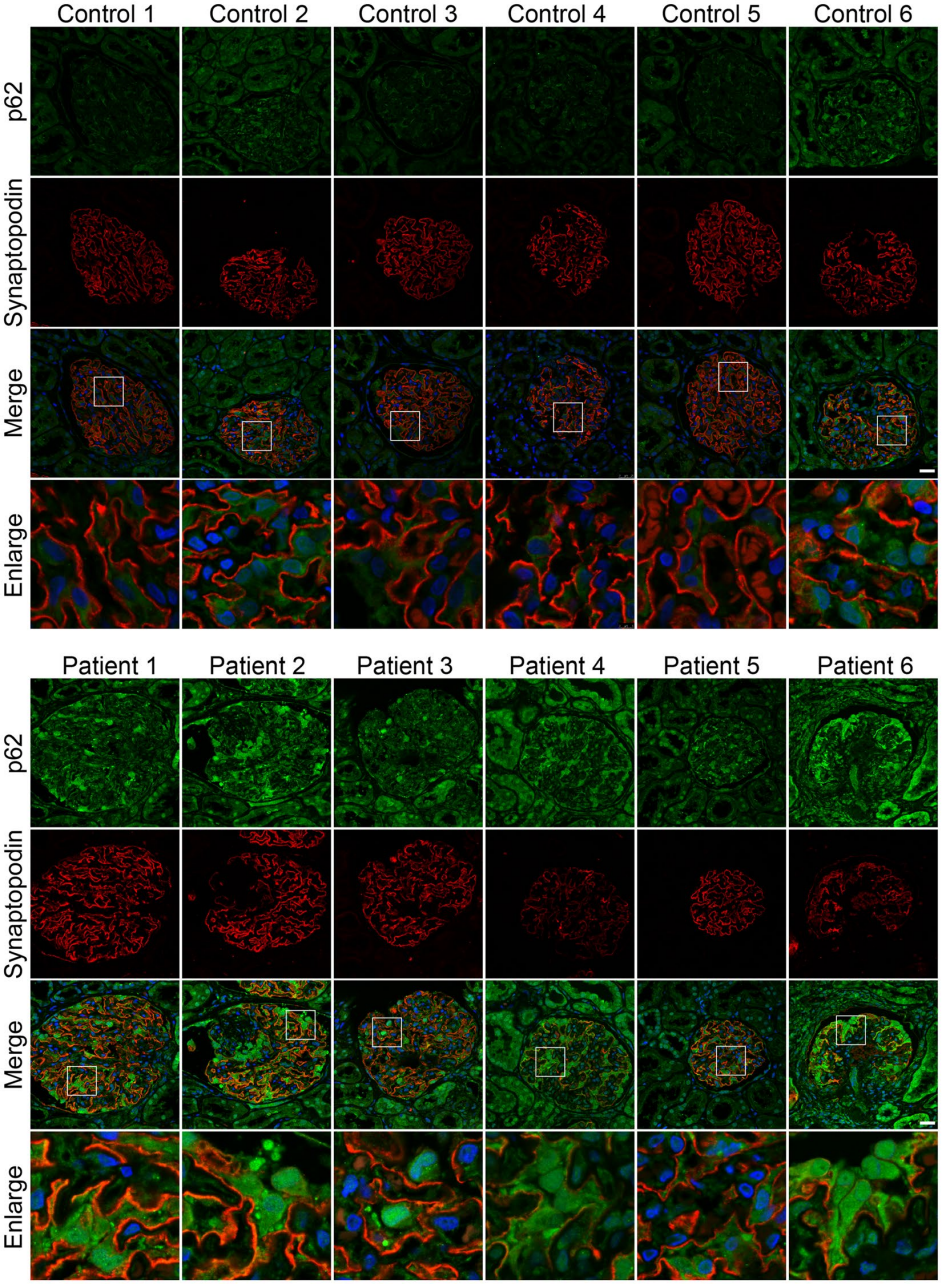
Autophagy impairment in podocytes of patients with idiopathic membranous nephropathy (IMN). Immunofluorescent analysis showing large accumulations of p62 protein (green) in podocytes of patients with IMN. The podocytes in glomeruli were identified by immunofluorescent double-labeling with synaptopodin (red), a podocyte foot process-specific protein. The nuclei were stained with DAPI (blue). Images were obtained using a confocal microscope. Scale bar, 25 μm.

Since p62/SQSTM1 protein is a specific target of autophagy degradation, the intracellular accumulation of this protein is indicative of insufficient autophagy^18^. Therefore, we next examined expression of the autophagy substrate p62 to determine whether the observed accumulation of autophagic vacuoles was a sign of autophagic induction and/or impaired autophagic degradation. The results showed very low p62 expression in the podocytes of controls with markedly enhanced p62 expression levels in the podocytes of most of the IMN patients (Fig. 2). Also, the more strong unspecific background staining possibly occurred in the renal tissues from patients with IMN. These data indicate that autophagic vacuole accumulation is at least partially attributed to impaired autophagic degradation.

### C5b-9 inhibits autophagy activity in cultured podocytes

**Figure 3:**
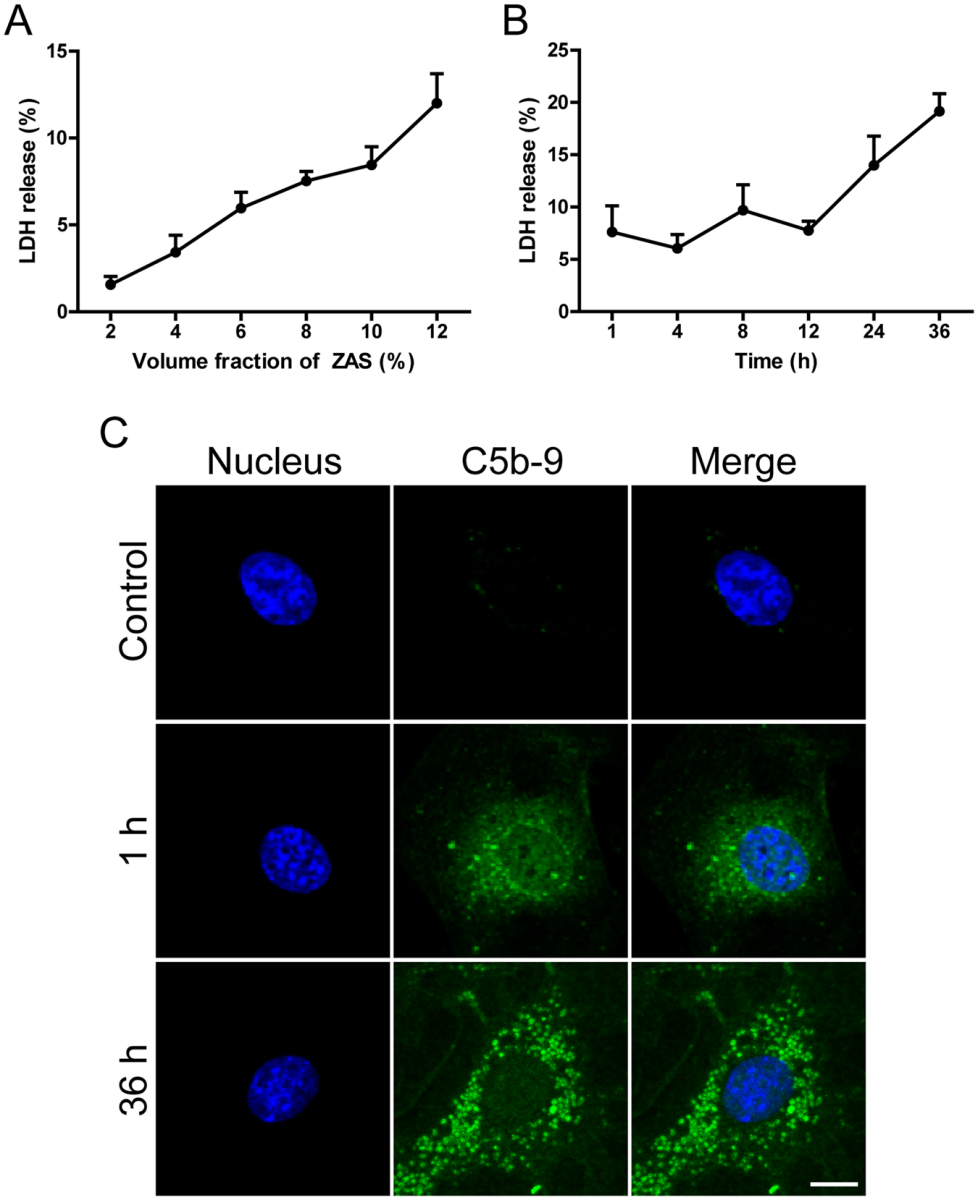
Assembly of sublytic C5b-9 on podocytes after exposure to zymosan activation serum (ZAS). (**A**,**B**) Lactate dehydrogenase (LDH) assay, quantified by the optical density of the supernatant at 450 nm. The release rate of LDH from ZAS-treated podocytes gradually increased in a volume fraction-dependent manner as of the 1-h time point. After exposure of podocytes to ZAS at 10% dilution, the release rate of LDH increased by less than 10% within 12 h, and then further increased at 24 h to 36 h. The data represent mean ± SD. (**C**) Immunofluorescent showing a lot of C5b-9-positive puncta in ZAS (10%)-treated podocytes from 1 h to 36 h, while rare in HIS (10%)-treated podocytes. The nuclei were stained with DAPI (blue). Images were obtained using a confocal microscope. Scale bar, 25 μm.

**Figure 4:**
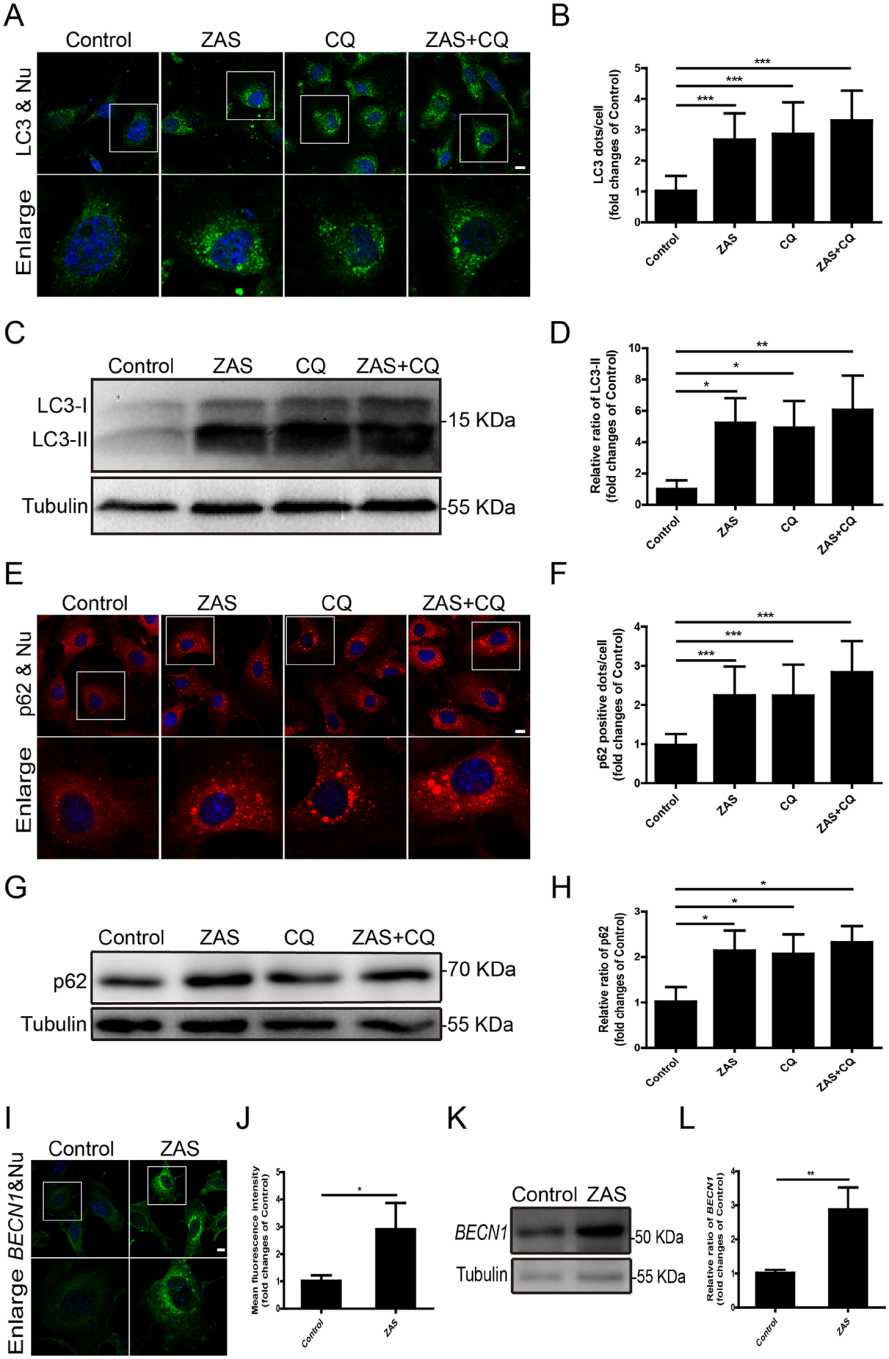
Inhibition of autophagy in podocytes after exposure to zymosan activation serum (ZAS). Podocytes were treated with heat-inactivated human serum (HIS) or ZAS with or without chloroquine (CQ, 10 μM) for 4 h. (**A**,**B**) Representative fluorescence images of LC3 (green) and DAPI (blue) staining revealed the expression of LC3 puncta was significantly increased after exposure to ZAS when compared with control, but not further elevated by CQ addition. (**C**,**D**) Immunoblotting analysis showed that ZAS significantly increase the expression of LC3-II protein, whereas the concomitant use of ZAS and CQ did not induce a further increase in LC3-II level. (**E**,**F**) Representative fluorescence images of p62 (red) and DAPI (blue) staining revealed much more p62 puncta was accumulated in podocytes after exposure to ZAS with or without CQ when compared to control. (**G**,**H**) Immunoblotting analysis showed that the expression of p62 was significantly higher in podocytes treated with ZAS, CQ or ZAS+CQ than that in control. (**I**,**J**) Representative fluorescence images of *BECN1* (green) and DAPI (blue) staining revealed the expression of *BECN1* was increased in podocytes after exposure to ZAS at 4 h. (**K**,**L**) Immunoblotting analysis showed that the protein level of *BECN1* was markedly enhanced in podocytes after exposure to ZAS when compared to control. All the fluorescence images were obtained using a confocal microscope. Scale bar, 10 μm. The data represent the fold-change relative to the control cells and are shown as mean ± SD. *P < 0.05, **P < 0.01, ***P < 0.001.

To mimic the process of podocyte lesion in IMN, the mouse podocyte cell line MPC-5 was treated with a medium containing different concentrations of zymosan activation serum (ZAS), since zymosan could promote rapid C3 cleavage by the alternative pathway to initiate the formation of C5b-9^14–16^. As the control, the LDH assay was performed at different time points after exposure to heat-inactivated human serum (HIS) at 10% dilution, which showed that LDH release fluctuated, but stayed at relatively low levels (data not shown). Interestingly, ZAS could enhance the release of lactate dehydrogenase (LDH) in a dose-dependent manner (Fig. 3A). And ZAS at 10% dilution could increase the level of LDH release from podocytes by less than 10% within 12 h (Fig. 3B). Therefore, this level was used to generate the sublethal dose of C5b-9 to induce podocyte injury and establish a model of IMN *in vitro* ^19^. As expected, the number of C5b-9-positive puncta increased from 1 h to 36 h after podocyte exposure to ZAS (Fig. 3C). We subsequently assessed the autophagic vacuoles in the sublytic C5b-9 membrane attack mode of podocytes, and found that ZAS induced a significant increase in LC3-II protein at 4 h. Similar results were obtained at 36 h (data not shown). LC3-II turnover was next tested in the presence and absence of chloroquine (CQ), which inhibits the fusion between autophagosomes and lysosomes and blocks lysosomal degradation via increasing the intralysosomal pH^20^ (Fig. 4A–D). Immunofluorescent staining showed that ZAS pretreatment increased the numbers of LC3 puncta in podocytes, which was not further elevated by CQ addition at 4 h (Fig. 4A,B). Consistently, no additional significant increase in LC3-II was observed in the CQ plus ZAS-treated group compared to the ZAS only-treated group, as determined by the western blot assay, although an increasing trend was observed at 4 h (Fig. 4C,D). In addition, LC3-II turnover was not observed at 36 h (data not shown). The positive aggregates and protein level of p62 were enhanced after exposure to ZAS (Fig. 4E–H), which is likely an indicator of autophagy inhibition. The upstream molecules of the autophagy pathway were also studied by evaluating the expression of Beclin 1 (*BECN1*), an essential protein in the onset of autophagy^21^. We found that the immunofluorescent intensity and protein level of *BECN1* were markedly enhanced after exposure to ZAS (Fig. 4I–L). These data indicate that LC3-II could not be transported to the lysosomes for degradation and that the autophagic pathway is blocked even in the face of autophagic induction via exposure of podocytes to C5b-9.

**Figure 5:**
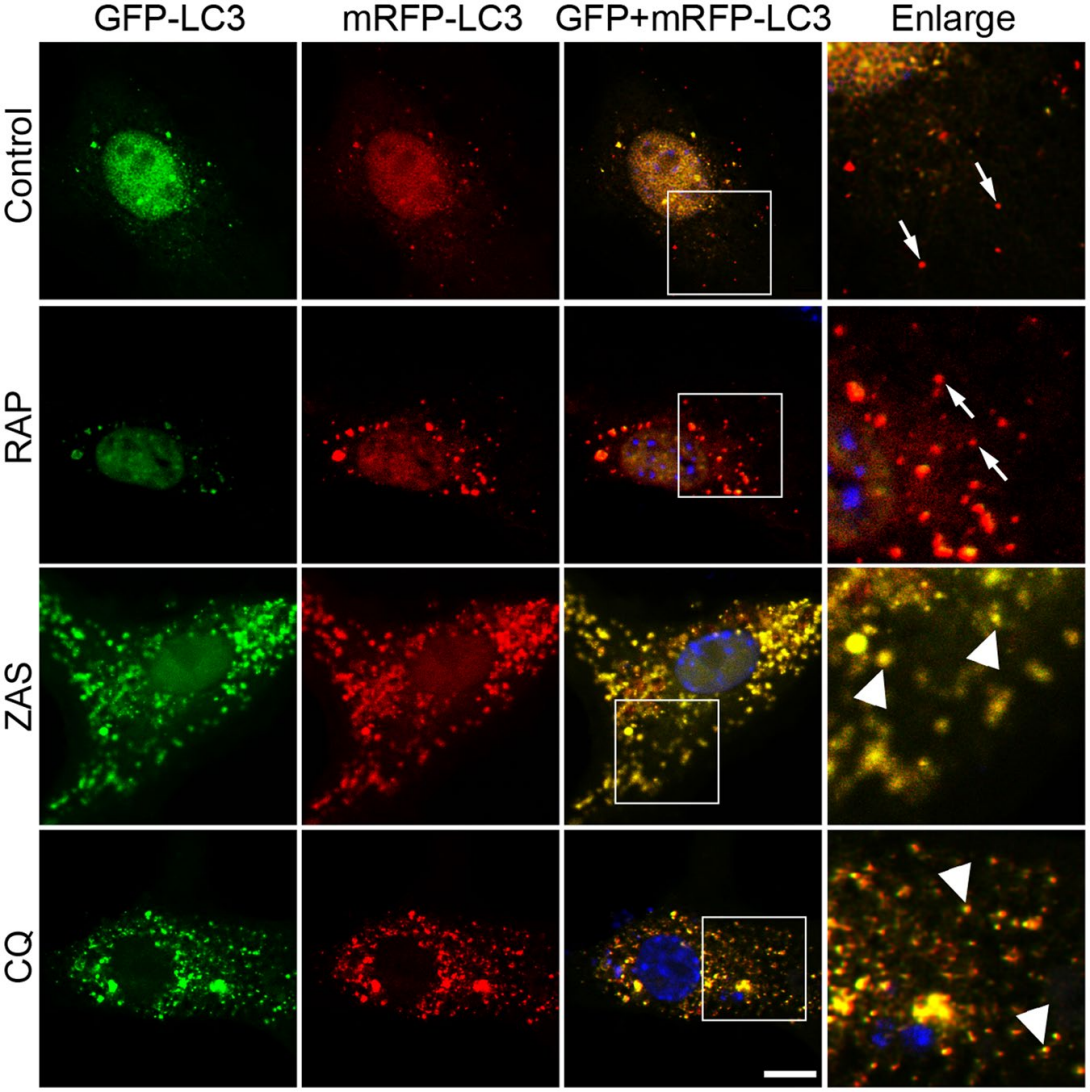
Zymosan activation serum (ZAS) blocks autophagic pathway by inhibiting autolysosome formation. Autophagic flux analysis with tandem mRFP-GFP fluorescent-tagged LC3 (tfLC3) plasmid, which showed GFP and mRFP fluorescence signals (yellow) before the autophagosomes fusion with lysosomes, whereas exhibited only the mRFP fluorescence signal (red) after autophagosome and lysosome fusion to become autolysosome due to GFP fluorescence signal (green) quenched in acid environment. After transfection, the cultured podocytes were subjected to heat-inactivated human serum (HIS), rapamycin (RAP, 10 μM), ZAS, or chloroquine (CQ, 10 μM) treatment for different times, fixed, and analyzed by confocal microscopy. The positive control podocytes treated with RAP displayed much more autolysosomes (free red dots) in the cytoplasm compared with the HIS treated control podocytes. However, ZAS induced accumulation of autophagosomes exhibiting increased yellow puncta but almost no free red dots in the cytoplasm from 4 to 36 h. The number of yellow puncta (autophagosomes) was also significantly increased in the presence of CQ. The representative images at 36 h were shown in this study. Arrowheads indicate autophagosomes. Arrows indicate autolysosomes. Scale bar, 10 μm.

Finally, the autophagosomes and autolysosomes were separately assessed in podocytes transfected with a monomeric red fluorescent protein (mRFP)-green fluorescent protein (GFP) tandem fluorescent-tagged LC3 (tfLC3) plasmid. Conventionally, the GFP (green) signal is quenched in the acidic condition of the normal lysosome lumen, whereas the mRFP (red) signal is more stable. Therefore, the dots emitting both green and red fluorescence (yellow) represent autophagosomes that have not been acidified by lysosomes. In contrast, the vacuoles with a free red signal correspond to autolysosomes. As shown in Fig. 5, some autophagosomes and autolysosomes were clearly detected after transfection. Subsequently, the cells were treated with rapamycin, the inhibitor of mammalian target of rapamycin (mTOR), which increases autophagic flux by promoting autophagosome formation. Indeed, rapamycin could induce a significant increase in both autophagosomes and autolysosomes, indicating autophagic activation. However, after ZAS treatment, the autophagosomes significantly increased, whereas almost no autolysosomes were detected in the podocytes, suggesting that many of the autophagosomes were not degraded by lysosomes. Next, we treated cells with lysosomal inhibitor CQ. Only autophagosomes, but not autolysosomes, were increased after exposure to CQ, similar to the action of ZAS. All these phenomena persisted from 4 h to 36 h *in vitro*. These data indicate that C5b-9 results in the inhibition of autophagosome degradation.

**Figure 6:**
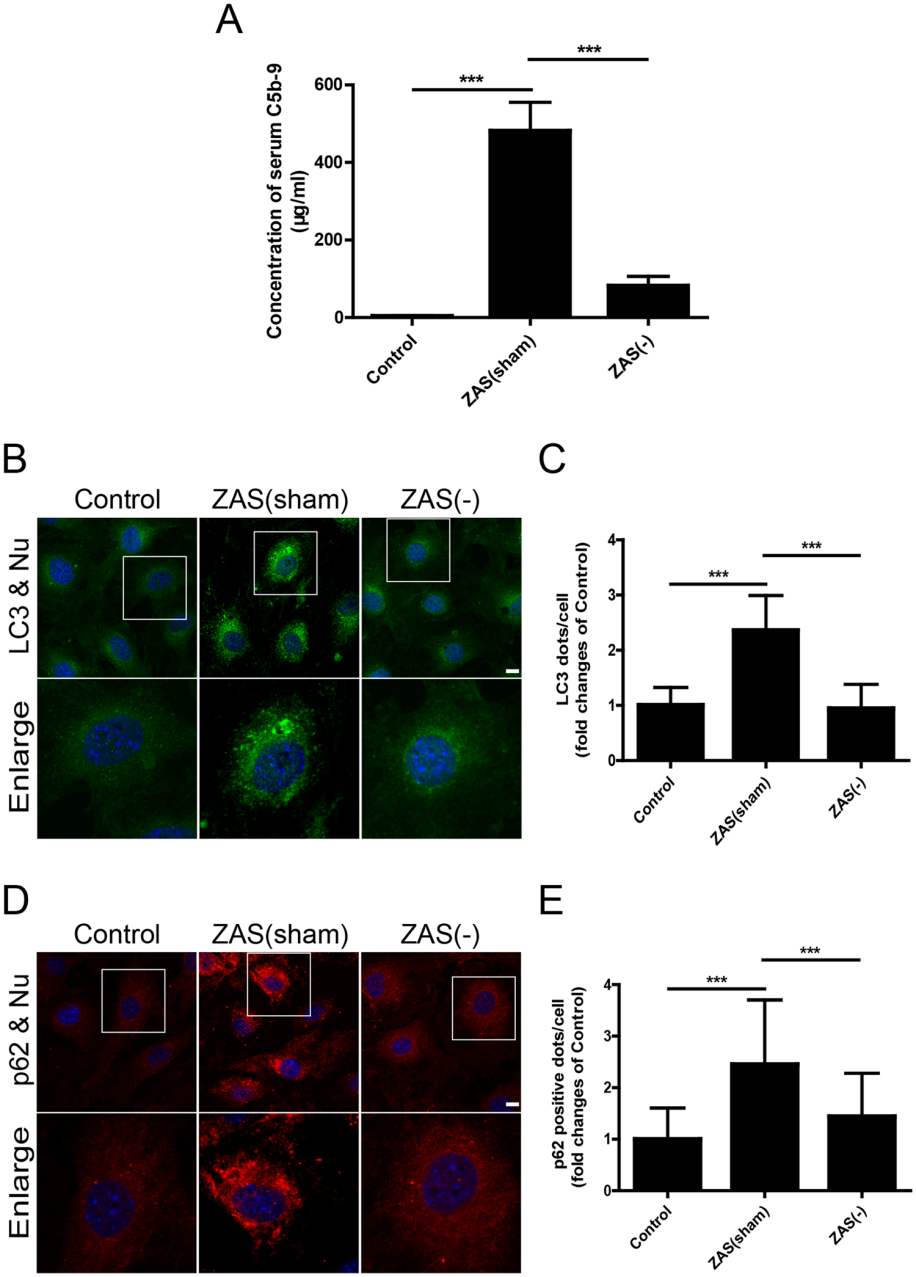
C5b-9-dependent autophagic changes after exposure of podocytes to zymosan activation serum (ZAS). (**A**) The results from enzyme-linked immunosorbent assay showed that the C5b-9 level of ZAS(sham) was higher than that in heat-inactivated human serum (HIS, as the control). However, the level of C5b-9 in ZAS(−) was significantly lowered compared to ZAS(sham). ZAS(−) indicated that C5b-9 was depleted in serum via incubation with anti-human C5b-9 monoclonal antibody for the indicated time period. ZAS(sham) indicated samples of ZAS prepared by an identical method. (**B**,**C**) Representative fluorescence images of LC3 (green) and DAPI (blue) staining revealed the number of LC3-positive puncta was significantly increase in podocytes after exposure to ZAS(sham) for 4 h but was abolished by ZAS(−). (**D**,**E**) Representative fluorescence images of p62 (red) and DAPI (blue) staining showed p62-positive aggregates in ZAS(sham)-treated podocytes, which was not observed in ZAS(−)-treated podocytes at 4 h. All the fluorescence images were obtained using a confocal microscope. Scale bar, 10 μm. The data are mean ± SD. ***P < 0.001.

To further verify the specific C5b-9-dependent effects in podocytes, a co-immunoprecipitation experiment was used to deplete C5b-9 from the serum. The C5b-9 level of the ZAS(sham) group (serum samples prepared with an identical method without antibody addition) was higher than that in normal human serum. However, the level of C5b-9 in the ZAS(−) group (in which C5b-9 was depleted by immunoprecipitation) was significantly lower compared to that of the ZAS(sham) group (Fig. 6A). And ZAS(−) could significantly reduce the C5b-9-positive puncta on podocyte membrane compared with ZAS(sham) (data not shown). In line with the results shown in Fig. 4, the numbers of LC3- and p62-positive puncta were significantly increased in ZAS(sham)-treated podocytes in comparison with those of the control, whereas ZAS(−) failed to enhance the numbers of LC3-positive puncta and p62-positive aggregates (Fig. 6B–E), indicating that the inhibitory effects of ZAS on autophagic activity are C5b-9-dependent.

### C5b-9-triggered autophagy inhibition induces podocyte injury

**Figure 7:**
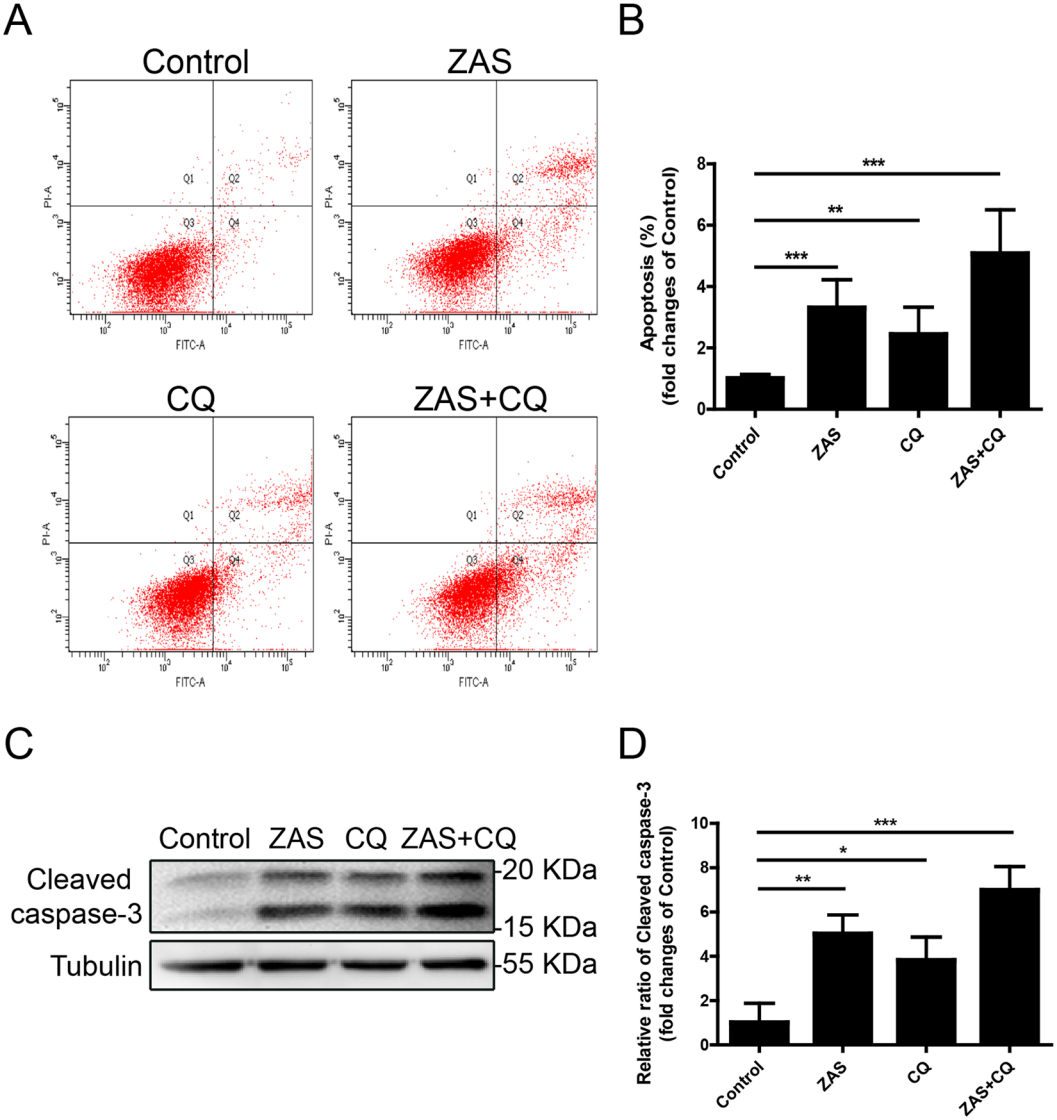
Podocyte injury triggered by zymosan activation serum (ZAS) induced autophagy inhibition. (**A**,**B**) Flow cytometric analysis showed enhanced apoptosis rates in podocytes after ZAS treatment with or without CQ (10 μM) for 36 h. (**C**,**D**) Immunoblotting analysis revealed an increase of cleaved caspase-3 protein in podocytes treated with ZAS with or without CQ (10 μM). The data represent the fold-change relative to the control cells and are shown as mean ± SD. *P < 0.05, **P < 0.01, ***P < 0.001.

We next studied whether ZAS-induced autophagy inhibition contributed to the formation of podocyte lesions. The extent of podocyte apoptosis was measured after double-staining with fluorescein isothiocyanate (FITC)-annexin V and propidium iodide (PI) followed by flow cytometric analysis. The addition of CQ or ZAS markedly enhanced podocyte apoptosis rates when compared with those of the control group (Fig. 7A,B). The ZAS-induced apoptosis was further confirmed by immunoblot analysis of cleaved caspase-3 at 36 h (Fig. 7C,D). These data suggest that one of the important mechanisms underlying podocyte injury in IMN is likely autophagy inhibition induced by C5b-9.

### An impaired lysosome is responsible for autophagy inhibition after exposure to C5b-9

**Figure 8:**
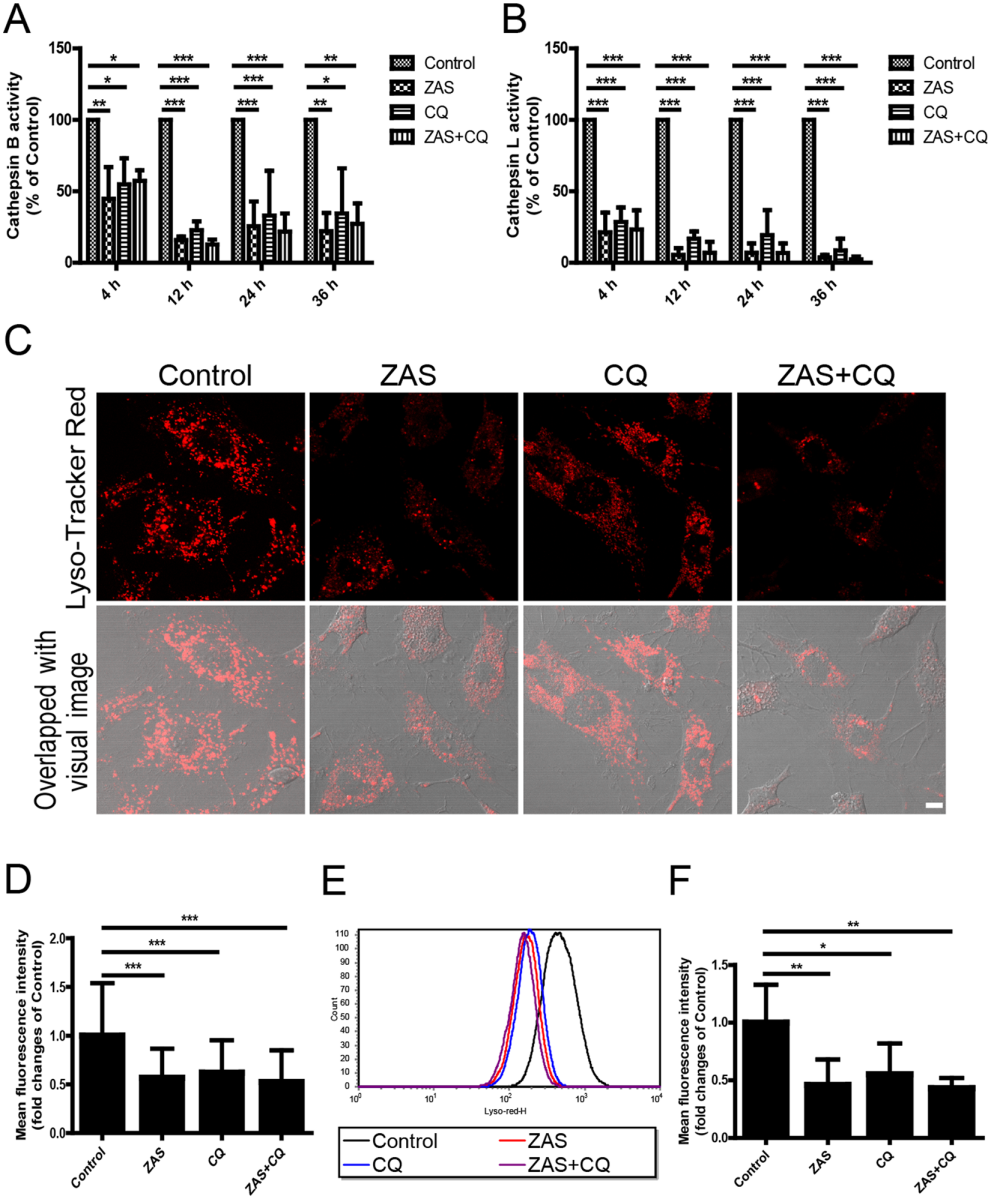
Zymosan activation serum (ZAS) induces decreased enzymatic activity and impaired acidification of lysosomes in podocytes. (**A**,**B**) Markedly decreased proteolytic activity of cathepsin B and cathepsin L was observed in podocytes after exposure to ZAS with or without chloroquine (CQ, 10 μM) from 4 h to 36 h using a fluorescence-based assay kit. (**C**,**D**) Impaired acidification of lysosomes was found by staining with Lyso-Tracker Red, an indicator of acidic intracellular compartments, in podocytes after exposure to ZAS with or without CQ (10 μM) for 4 h. Images were obtained using a confocal microscope and the mean fluorescent intensity of Lyso-Tracker Red in podocytes was quantified using Leica confocal software. Scale bar, 10 μm. (**E**,**F**) Representative histogram obtained from Lyso-Tracker Red staining followed by flow cytometric analysis indicated the decreased Lyso-Tracker Red signals and mean fluorescent intensity in podocytes after exposure to ZAS with or without CQ (10 μM) for 4 h. The data represent the fold-change relative to the control cells and are shown as mean ± SD. *P < 0.05, **P < 0.01, ***P < 0.001.

Lysosomes are the major digestive organelles in eukaryotic cells, playing a particularly essential role in the degradation of autophagosome contents at the end stages of autophagy^22^. Therefore, we next investigated the activity of lysosomal proteolytic enzymes in the sublytic C5b-9 membrane attack mode of podocytes. Measurement of the proteolytic activity showed a significant decrease in cathepsin B activity after exposure of podocytes to ZAS, CQ, or ZAS plus CQ at 4, 12, 24, and 36 h compared to that of the control. Similar results were obtained when assessing cathepsin L activity (Fig. 8A,B). Acidification of lysosomes is essential for most enzymes to exert their maximum activity; thus, the elevation of lysosomal pH may impair the activity of hydrolytic enzymes and elicit the declined degradability of the lysosome^23^. We used Lyso-Tracker Red to label and track the acidic intracellular compartments (lysosomes) in live cells, which showed that the numbers of punctate red fluorescence (lysosomes) clearly decreased following treatment with ZAS, which is similar to the action of CQ. In addition, the numbers of punctate lysosomes did not further decline by the addition of CQ together with ZAS as assessed by immunofluorescence staining (Fig. 8C,D). In parallel, the Lyso-Tracker Red signal (mean fluorescent intensity) also decreased after exposure to ZAS, CQ, or ZAS plus CQ for 4 h as determined by flow cytometry analysis (Fig. 8E,F). This phenomenon persisted from 4 h to 36 h *in vitro* (data not shown at the other time points). Together, these data indicate that C5b-9 abolishes the acidification of lysosomes, leading to lysosomal dysfunction.

**Figure 9:**
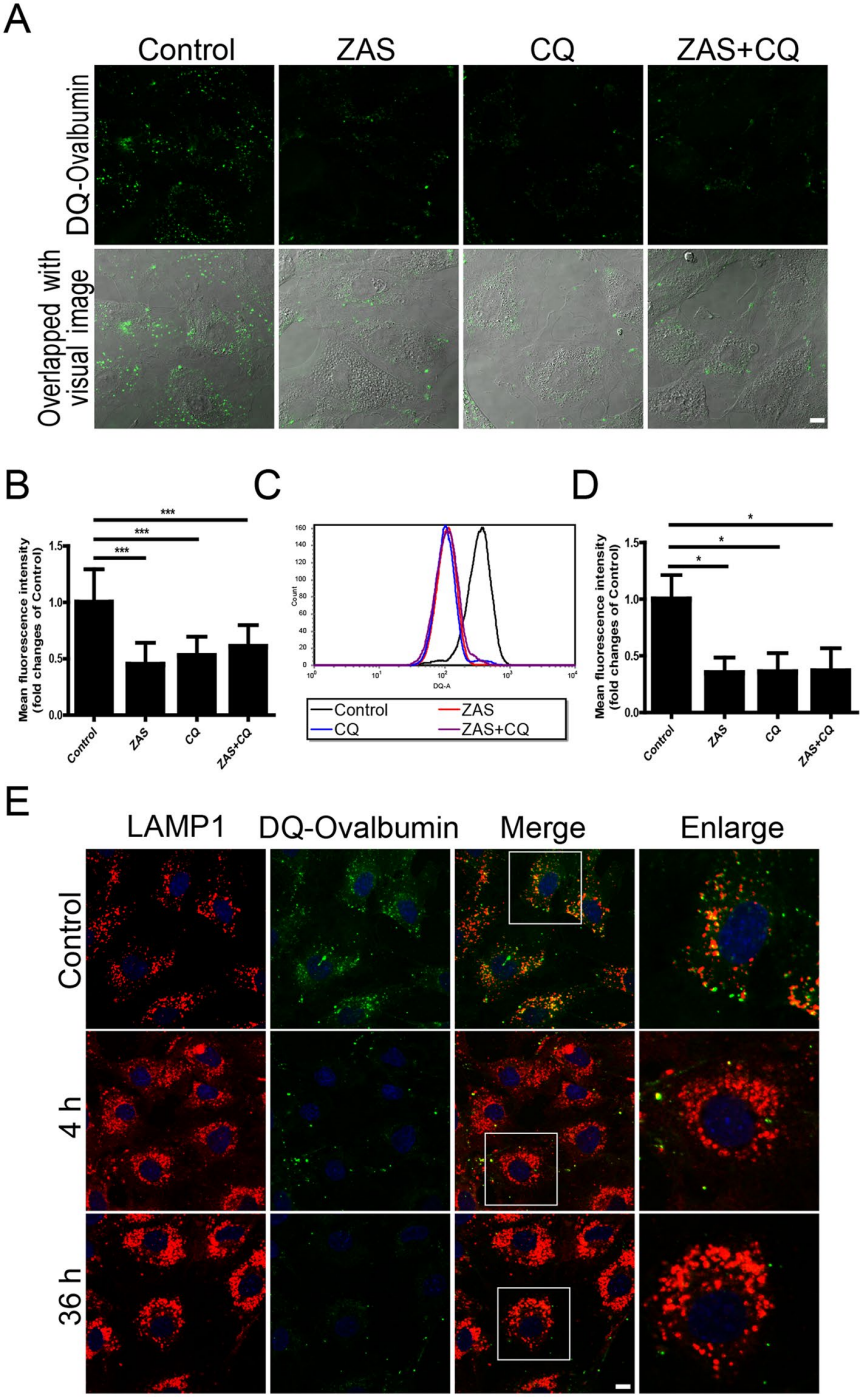
Zymosan activation serum (ZAS) induces declined degradability of lysosomes in podocytes. (**A**,**B**) Significantly declined proteolytic degradation of lysosomes was demonstrated by staining with DQ-ovalbumin, a self-quenched substrate for proteases, in podocytes after exposure to ZAS, chloroquine (CQ, 10 μM), or ZAS plus CQ (10 μM) for 4 h. (**C**,**D**) Representative histogram obtained from DQ-ovalbumin staining followed by flow cytometric analysis indicated the decreased DQ-ovalbumin signals and mean fluorescent intensity in podocytes after exposure to ZAS with or without CQ (10 μM) for 4 h. (**E**) Immunofluorescent analysis of LAMP1-labeled lysosomes (red) and the dequenched DQ-ovalbumin vesicles (green) showed the lysosomal degradation of DQ-ovalbumin was compromised in podocytes after exposure to ZAS from 4 h to 36 h. Images were obtained using a confocal microscope and the mean fluorescent intensity of DQ-ovalbumin in podocytes was quantified using Leica confocal software. Scale bar, 10 μm. *P < 0.05, ***P < 0.001.

**Figure 10:**
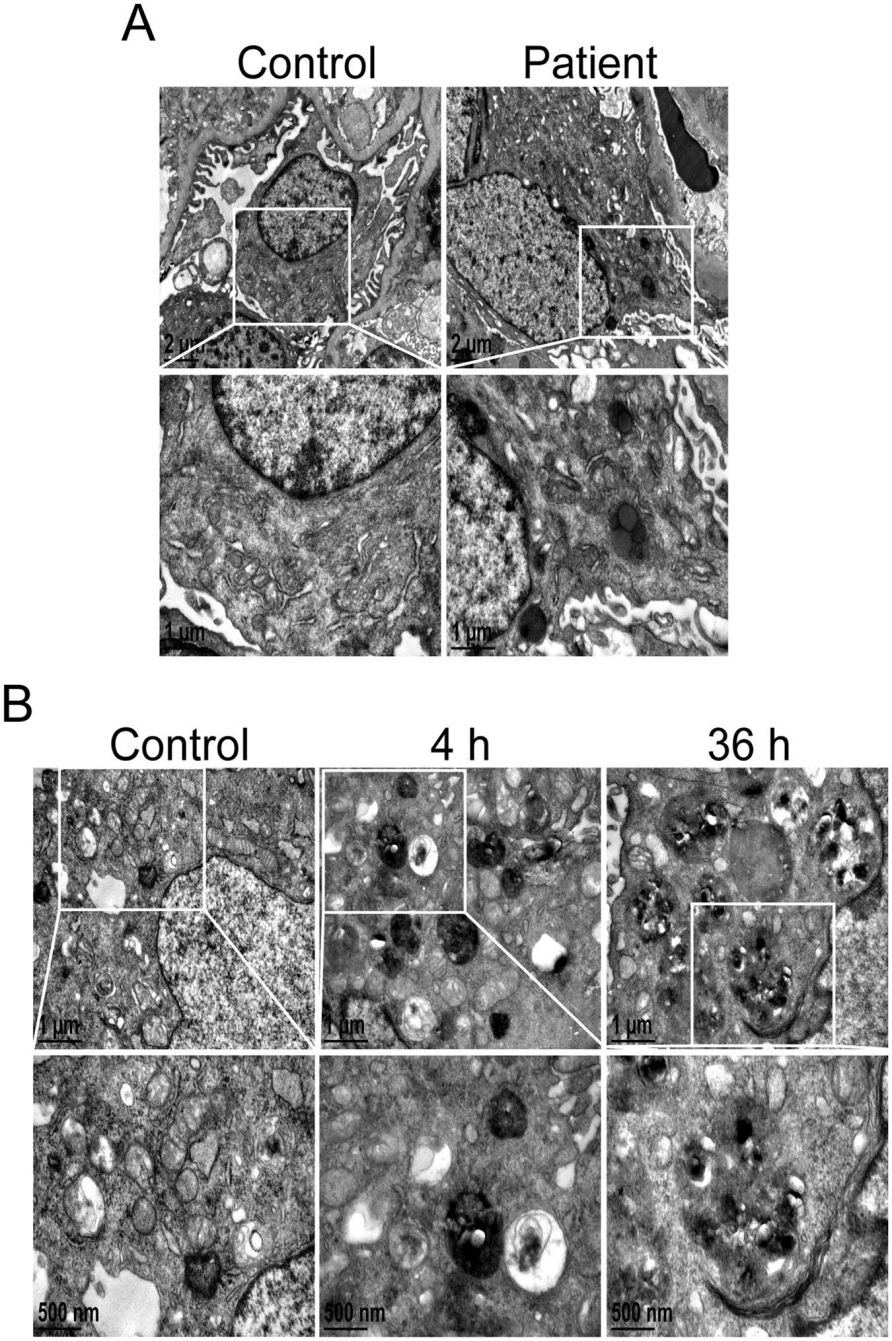
Ultrastructural images of lysosomes and autophagic vacuoles in podocytes from patients with idiopathic membranous nephropathy (IMN) and zymosan activation serum (ZAS)-induced C5b-9 membrane attack models. Representative electron microscope images showing impaired lysosomes as dark irregular structures in podocytes of kidney biopsy specimens from patients with IMN (**A**), and in podocytes after exposure to ZAS at 4 h, as well as lysosomes engulfed by autophagic vacuoles at 36 h (**B**).

To further evaluate the efficiency of lysosome-mediated proteolytic degradation in podocytes, a self-quenched substrate for proteases, DQ-ovalbumin, was used^24^. The mean fluorescent intensity of the DQ-ovalbumin signal was significantly lower in ZAS-, CQ-, or ZAS plus CQ-treated cells than in controls at 4 h, as assessed by fluorescence confocal microscopy (Fig. 9A,B) and flow cytometry (Fig. 9C,D); this phenomenon persisted until 36 h (data not shown). Lysosome-associated membrane protein-1 (LAMP1) was utilized to study the relationship between lysosomal degradation and dequenched DQ-ovalbumin. As shown in Fig. 9E, a large number of dequenched DQ-ovalbumin dots were co-localized with LAMP1-positive puncta in vehicle-treated podocytes, indicative of the lysosomal degradation of DQ-ovalbumin. However, along with the occurrence of irregular and larger LAMP1 granules, few DQ-ovalbumin vesicles were detected in the podocytes after exposure to ZAS for 4 h, which remained apparent at 36 h. Transmission electron microscopy (TEM) analysis also revealed impaired lysosomes present as dark irregular structures in the podocytes from IMN patients as well as in ZAS-treated cells (from 4 to 36 h), which were not detectable in controls (Fig. 10A,B). In addition, some damaged lysosomes were likely engulfed by autophagic vacuoles to form lysophagy after exposure of podocytes to ZAS for 36 h. These results suggest that lysosomal degradability is compromised after exposure to C5b-9.

### Lysosomal membrane permeabilization is triggered by C5b-9 in podocytes

**Figure 11:**
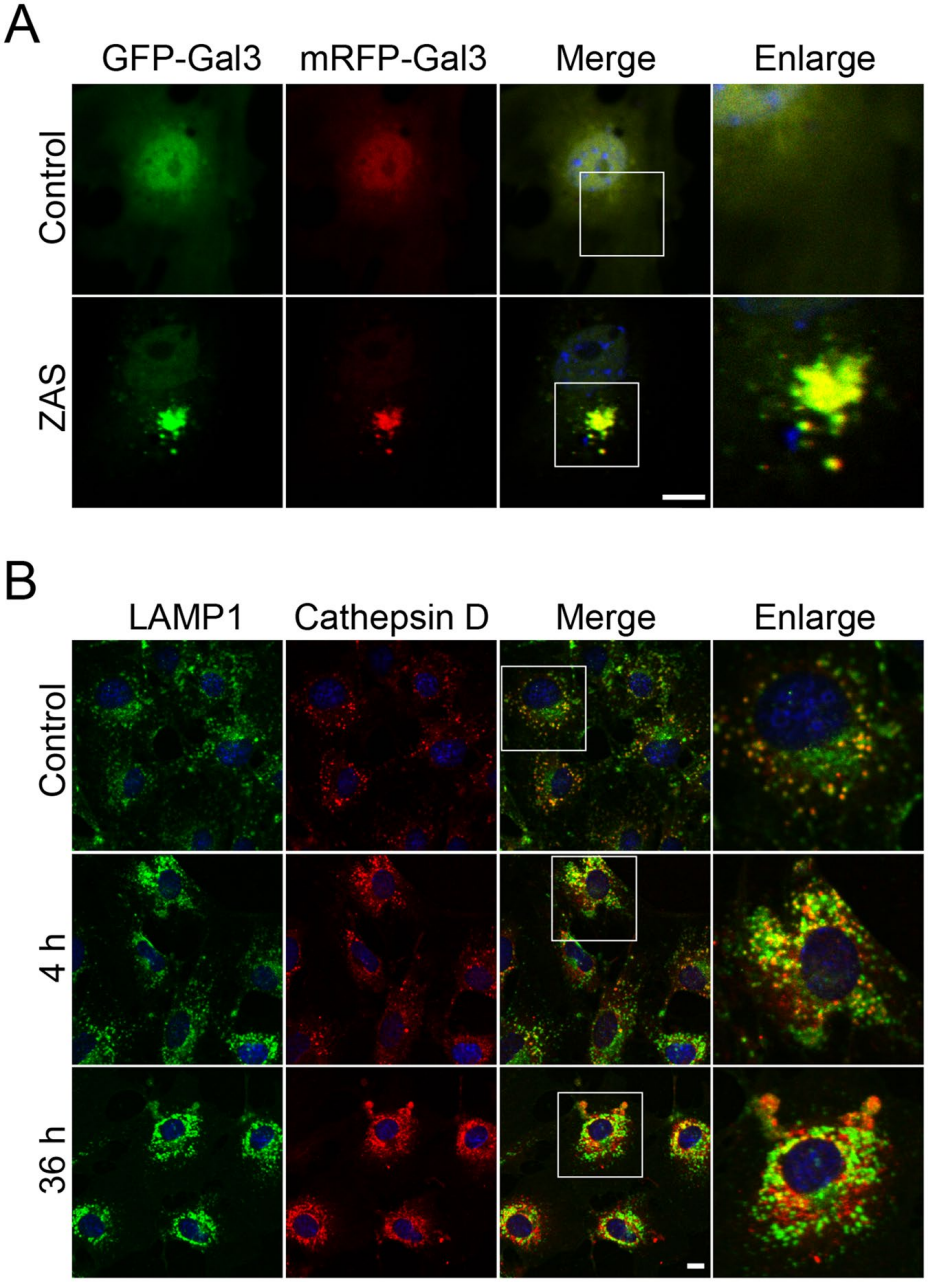
Lysosomal membrane permeabilization triggered by zymosan activation serum (ZAS). (**A**) The cultured podocytes were transfected with tandem fluorescent-tagged Galectin-3 (tfGal3) plasmid, which can accumulate inside the ruptured lysosomes, followed by treatment with heat-inactivated human serum (HIS) or ZAS for 4 h. Representative fluorescence images show that tfGal3 puncta (mRFP^+^GFP^+^) were clustered in ZAS-treated podocytes, indicating membrane rupture in the lysosomes. (**B**) Immunofluorescent double-staining with LAMP1 and cathepsin D in podocytes after exposure to HIS (control) and ZAS for 4 h or 36 h. The red immunofluorescence shows the leakage of cathepsin D (red) from the lysosomes in the cytoplasm accompanied by irregular LAMP1 (green) granules at 36 h. Scale bar, 10 μm.

Our previous studies revealed that lysosomal membrane permeabilization is a primary cause of lysosomal dysfunction in renal tubular epithelial cells under certain conditions such as exposure to advanced glycation end products^25^ or urinary proteins^26^. To investigate lysosomal membrane permeabilization, we used the tandem fluorescent-tagged Galectin-3 (tfGal3) plasmid as a probe to detect the damaged lysosomes. Upon ZAS treatment, tfGal3 formed a cluster with mRFP^+^GFP^+^ puncta in podocytes, whereas almost no tfGal3 puncta were observed in the control. This finding indicates that lysosome rupture occurs in the sublytic C5b-9 membrane attack model of podocytes, since the tfGal3 accumulated inside the ruptured lysosomes (green- and red-positive dots), but localized diffusely into the cytoplasm under normal conditions (Fig. 11A). Moreover, the cells were double-stained with cathepsin D and LAMP1. In vehicle-treated podocytes, LAMP1-immunoreactive granules were regularly distributed in the cytosol and most of them were co-localized with perinuclear cathepsin D, which displayed a fine and granular distribution pattern. However, the irregular and larger cytoplasmic LAMP1 granules were detected in podocytes after exposure to ZAS for 4 to 36 h, accompanied by dispersed cathepsin D expression (Fig. 11B). This finding indicates that lysosomal membrane permeabilization occurs in the sublethal C5b-9 membrane attack mode of podocytes, which may contribute to lysosomal dysfunction.

## Discussion

Various studies in animal models and patients have confirmed a mechanism of podocyte injury involving the complement-induced accumulation of misfolded proteins, accompanied by a change in the metabolic pathway^27, 28^. Therefore, the essential protein degradation pathway of autophagy is likely to be one of the most potent cytoprotective mechanisms occurring in podocytes. In the present study, impaired autophagy was confirmed in podocytes from patients with IMN, characterized by the accumulation of p62 and accompanied by an increase in autophagic vacuoles. However, a higher background likely occurred in the LC3 and p62 staining in renal tissues from patients with IMN, in spite of using different specific antibodies and methods (data not shown). What results in the strong background staining? Is the background staining an unspecific staining? All the issues need to be further studied in the future. **A**utophagy is a very complicated process, including autophagy induction, autophagosomes formation, the fusion of autophagosomes and lysosomes to become autophagolysosomes, and degradation of the vacuoles^21^. To determine the main nodes of this process that participate in autophagy inactivation, we investigated the detailed mechanism in a sublytic C5b-9 membrane attack model *in vitro*, since the increased expression of p62 and LC3 was found to be C5b-9-dependent. Interestingly, we found that *BECN1* expression was upregulated in the cellular model induced by C5b-9, suggesting that the upstream components of the autophagic pathway were not suppressed, since *BECN1* expression is a marker of autophagy induction^29^. When studying the downstream pathway of autophagy, the accumulation of autophagosomes but not autolysosomes was detected in C5b-9-treated podocytes. This finding suggests that the autophagosomes could not fuse with lysosomes to become autophagolysosomes. Furthermore, blockage of the lysosomal turnover of LC3-II was also observed in the podocyte model induced by the sublytic complement, suggesting that C5b-9 had disrupted the fusion of the autophagosome with the lysosome or that the lysosome had lost its degradative ability before addition of the lysosomal inhibitor. Therefore, the main characteristics of disrupted autophagy in podocyte are an increase in autophagosome formation and a decrease in autophagosome degradation in the progression of IMN.

It has been reported that autophagy inhibition leads to the aging-related loss of renal function and late-onset glomerulosclerosis^11^. Autophagy downregulation also contributes to the insulin resistance-mediated injury of podocytes in diabetic kidney disease^30^. Therefore, we subsequently evaluated the podocyte lesions after treatment with CQ to explore whether autophagy inactivation plays a role in the pathogenesis of IMN. We found that blocking the autophagic pathway resulted in an increased apoptosis of podocytes, which is similar to the action of complement activation. These data indicate that autophagy inactivation is one of the important mechanisms underlying the podocyte injury in IMN. Indeed, there is little doubt that the accumulation of damaged organelles and abnormal and misfolded proteins plays an important role in the lesions of podocytes. More importantly, autophagic stress, defined as a relatively sustained imbalance in which the rates of autophagosome formation exceed the rates of autophagosome degradation, was markedly enhanced by increasing autophagic induction and blocking the autophagic degradation process in the podocytes of patients with IMN. TEM analysis also showed that autophagic vacuoles contained different densities of undigested cargoes. The autophagy substrates included the damaged lysosomes as well as some dark irregular structures, which are characteristics of lysophagy^31^. Lysophagy is likely a typical feature of increased autophagic stress since it suggests the apparent dysfunction of lysosomes and then leads to the imbalance between autophagosome formation and degradation. Podocytes are probably killed directly when undergoing strong autophagic stress, as reported for some other cell types^32^.

It is well known that lysosomes are essential for the degradation of autophagosome^21^. Therefore, we next focused on lysosomal function to explore the processes occurring in lysosomes in the sublytic C5b-9 membrane attack model of podocytes. The lysosomal proteolysis capacity of cathepsin was decreased and lysosomal acidification became defective, indicative of lysosomal damage. More importantly, the lysosomal degradation of DQ-ovalbumin also declined, providing direct evidence of the blockage of the downstream pathway of autophagy. An autophagy-dependent dual role (protective or detrimental) has been identified in some nephropathies such as ischemia-reperfusion renal injury and protein overload-associated tubular epithelial cell lesions^26, 33, 34^, which was likely due to the difference between proper autophagic activation and an increase in autophagic stress. From this perspective, lysosomal function is probably a key to linking the dual actions of autophagy, since the autophagy-dependent adaptive response is mounted under the normal condition of lysosomes, while autophagic cell death is triggered in the abnormal state of lysosomes. Notably, in our study, lysosomal membrane permeabilization occurred in the sublytic C5b-9 membrane attack mode, characterized by cathepsin leakage from the lysosomes and irregular LAMP1 expression, as well as lysosomal membrane rupture. The occurrence of lysosomal membrane permeabilization is frequently associated with the overproduction of ROS^25, 26, 35^. Indeed, several studies have described the production of ROS after exposure to sublytic C5b-9^5, 36, 37^. However, further study is required to determine whether lysosomal membrane permeabilization is attributed to the increased oxidative stress after membrane attack by the complement. Nevertheless, lysosomal membrane permeabilization is one of the key factors to inactivate lysosomes in many other nephropathies, including diabetic kidney disease and urinary protein-associated disease, as reported in our previous studies^25, 26^. Unfortunately, despite substantial effort, there has been little progress made in developing strategies for preventing lysosomal membrane permeabilization.

**Figure 12:**
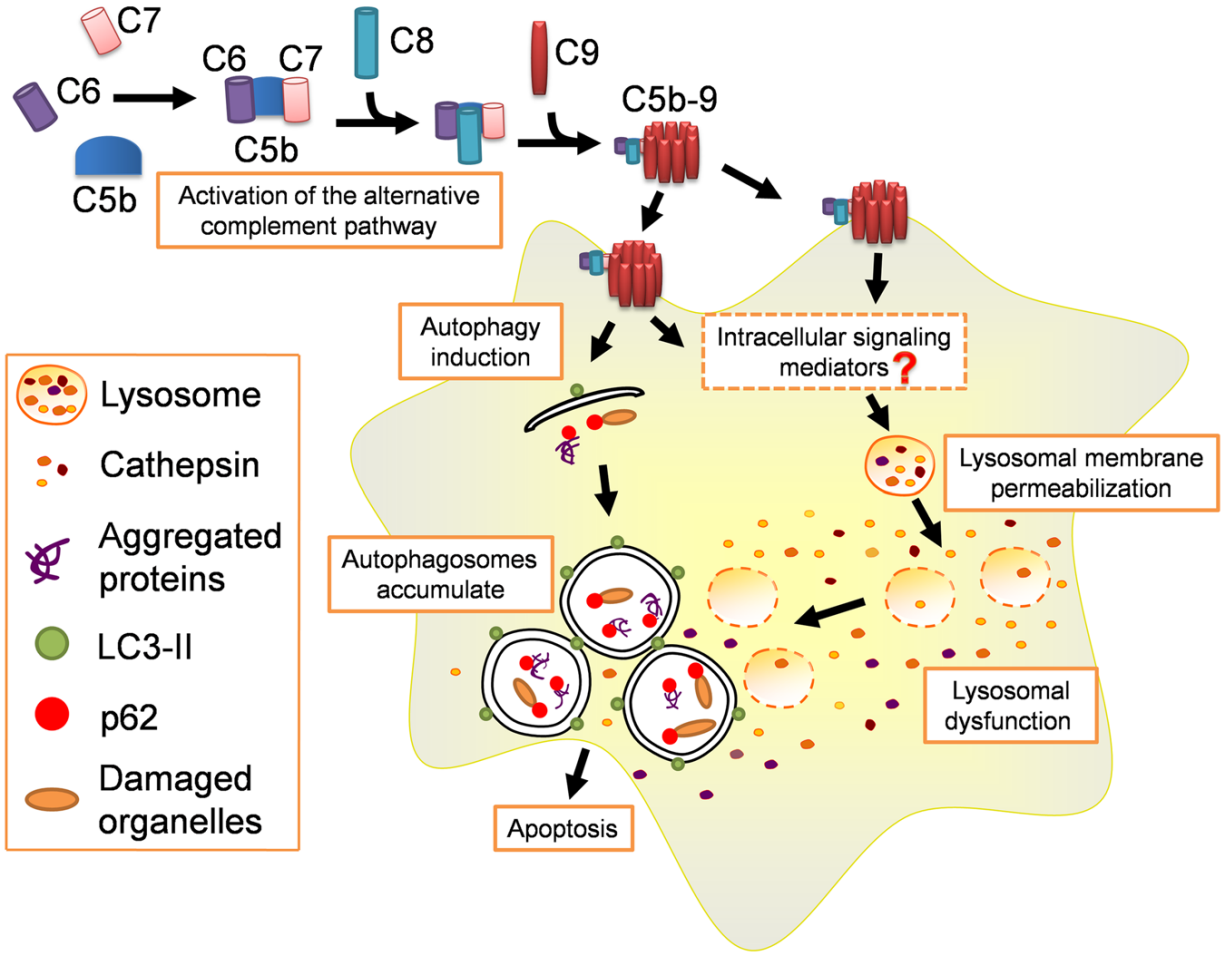
Schematic representation of C5b-9-induced lysosomal dysfunction that inactivates autophagy in podocytes. The sublytic C5b-9 membrane attack complex assembled via activation the alternative complement pathway increases autophagic induction, and also likely triggers intracellular signaling mediators to induce lysosomal dysfunction caused by lysosomal membrane permeabilization, resulting in the accumulation of autophagosomes and autophagic inactivation, which leads to podocyte injuries.

Taken together, our results show that lysosomal membrane permeabilization induced lysosomal dysfunction is triggered by the complement membrane attack complex C5b-9, which results in inhibition of the autophagic pathway to induce the podocyte injury in IMN (Fig. 12). Thus, restoring lysosome function provides a promising novel approach for treating IMN.

## Methods

### Patients

Seventeen kidney tissue specimens were obtained from biopsy-proven IMN patients with nephritic syndrome. Fourteen control kidney tissue specimens were obtained from patients underwent renal biopsy owing to asymptomatic slight proteinuria or hematuria, and subsequently excluded from glomerular disease by pathological examination. The experiments were conducted in accordance with the principles of the Declaration of Helsinki and were approved by the Institutional Review Board of the Affiliated Hospital of Guangdong Medical University. Written informed consent was obtained from all patients. All of the clinical data from 31 patients at the Affiliated Hospital of Guangdong Medical University were de-identified prior to analysis.

### Immunofluorescence of kidney specimens

Fixed kidney specimens embedded in paraffin were sectioned at a 3-μm thickness. The paraffin-embedded tissue sections were baked at 60 °C for 3 h, followed by deparaffinization and hydration according to standard procedures. The sections were subjected to antigen retrieval by boiling in citrate buffer (10 mM, pH 6.0) for 5 min and then treated with 0.3% H_2_O_2_ in methanol for 10 min. The sections were then incubated in 0.25% phosphate-buffered saline (PBS)-Tween for 1 h for permeabilization, and then placed in 3% bovine serum albumin (BSA) for 10 min to block non-specific protein–protein interactions before incubation with rabbit anti-C5b-9 (Abcam), mouse anti-synaptopodin (R&D Systems, Minneapolis, MN, USA), and rabbit anti-LC3B (MBL, Nagoya, Japan) or rabbit anti-p62 (MBL) overnight at 4 °C, followed by incubation with Alexa Fluor® 488 donkey anti-rabbit and Alexa Fluor® 594 donkey anti-mouse IgG (Invitrogen, Carlsbad, CA, USA). DAPI was used to stain the nuclei. The sections were examined by a TCS SP5 II confocal microscope (Leica Microsystems, Wetzlar, Germany).

### Cell culture and treatments

The immortalized mouse podocyte cell line MPC-5 was kindly provided by Dr. Wei Shi from Guangdong General Hospital and cultured in RPMI 1640 medium (Corning, New York, NY, USA) supplemented with 10% fetal bovine serum (Gibco, New York, NY, USA), 100 U/mL penicillin-streptomycin (Gibco), and 10–50 U/mL of mouse recombinant γ-interferon (Prospec, East Brunswick, NJ, USA) under a 5% CO_2_, 33 °C environment to induce proliferation. The podocytes were then transferred to a plate coated with type I rat-tail collagen (Gibco) under a 5% CO_2_, 37 °C environment without γ-interferon when they reached 60–70% confluence. For full differentiation in 8–12 days, the podocytes were used in the following experiments. To evaluate the effects of soluble C5b-9 on cultured podocytes, the sublytic C5b-9 membrane attack complex was established by using normal human serum as a complement source. To activate the complement, normal human serum was treated with zymosan (Sigma, St. Louis, MO, USA) as described by Ishikawa *et al*.^38^. MPC-5 cells were treated with media containing different concentrations of zymosan activation serum (ZAS) for 1, 4, 8, 12, 24, and 36 h, and heat-inactivated human serum (HIS) was used as a control. The volume fraction for ZAS to increase the LDH release by less than 10% was used as a sublethal dose to induce podocyte injury^19^. Ultimately, 100 µL/mL was utilized as the final concentration of HIS or ZAS. The cells were also exposed to a lysosomal inhibitor, chloroquine (CQ, 10 µM, Sigma) or ZAS plus CQ to block the autophagic pathway.

### LDH assay

The supernatant concentrations of LDH released from podocytes were measured after exposure to ZAS for different time points by assessing the values of the optical density at 450 nm using Lactate Dehydrogenase Activity Assay Kit (BioVision), according to the manufacturer instructions. The release rates of LDH in individual samples were calculated using the following formula: release rate (%) = [(experimental LDH activity value – background LDH activity value)/(maximum LDH activity value + experimental LDH activity value – background LDH activity value)] × 100%.

### Immunofluorescence staining of cultured podocytes

The podocyte samples were fixed with 4% paraformaldehyde for 15 min at room temperature, rinsed with PBS, and then permeabilized with 0.5% Triton X-100 in PBS. Non-specific binding was blocked with 5% BSA for 1 h at room temperature. To assess autophagy activity, the podocytes were incubated with rabbit anti-p62 (MBL), rabbit anti-LC3B, rabbit anti-LAMP1, mouse anti-cathepsin D, and mouse anti-*BECN1* (Abcam) overnight at 4 °C. After washing with PBS, the podocytes were labeled with Alexa Fluor® 488 donkey anti-rabbit or Alexa Fluor® 594 donkey anti-mouse IgG (Invitrogen). Images were obtained by a TCS SP5 II confocal microscope. The LC3 or p62 puncta/cell was determined from at least 30 cultured cells for each group blindly by two independent investigators.

### Immunoprecipitation method

C5b-9 was depleted in ZAS by immunoprecipitation as described by Ishikawa *et al*.^38^. An anti-human C5b-9 monoclonal antibody (Quidel, San Diego, CA, USA) was added to freshly prepared ZAS at a molar ratio of 1:1 and incubated first at 22 °C (2 h), then at 4 °C (24 h) while the samples were continuously rotated. Protein G Plus/Protein A Agarose Suspension (Millipore, Billerica, MA, USA) was added to the mixture and incubated for 3 h at 22 °C with constant rotation of the samples. Substrate-bound immune complexes were removed from the serum by centrifugation (3,000 × *g*, 10 min) and filtration (0.8 μm). Sham samples of serum were prepared by identical methods except that the antibody was not added. SC5b-9 was quantified by enzyme-linked immunosorbent assay (Quidel) in the treated sera.

### Transmission electron microscopy

Electron microscopy was performed as described previously^34^. In brief, kidney tissue specimens from patients and cells were fixed in 2.5% glutaraldehyde in 0.1 M sodium phosphate buffer, and then dehydrated in a graded ethanol series and embedded. Ultrathin sections were mounted on nickel grids. The samples were then stained and examined using a JEM-1400 electron microscope (JEOL, Tokyo, Japan).

### Lysosomal enzymatic activity assay

Detection of cathepsin B or cathepsin L activity in podocytes was carried out using a fluorescence-based assay kit (BioVision, Milpitas, CA, USA) according to the manufacturer’s instructions. In brief, after cleavage of the synthetic substrate by the cell lysate, the released fluorescence was quantified using an EnSpire multimode plate reader (PerkinElmer, Santa Clara, CA, USA) with an excitation filter of 400 nm and an emission filter of 505 nm.

### Lyso-Tracker Red uptake test

The acidification of lysosomes was determined with Lyso-Tracker Red (Invitrogen) according to the manufacturer’s instructions. Lyso-Tracker Red can form aggregates in lysosomes emitting red fluorescence in an acidic environment. In brief, for immunofluorescence, after being cultured on sterile culture dishes, each group of treated podocytes was incubated with 50 nM Lyso-Tracker Red for 15 min. The cells were then washed with PBS to remove any excess lysosomal marker, and then fixed with 4% paraformaldehyde for 10 min and visualized using a TCS SP5 II confocal microscope. The mean fluorescent intensity of the red-fluorescent Lyso-Tracker Red puncta in individual podocytes was calculated and presented as a histogram. For flow cytometric detection, after incubation with 50 nM Lyso-Tracker Red for 15 min, the podocytes were trypsinized and re-suspended in PBS. The fluorescent intensity value of Lyso-Tracker Red was quantified by flow cytometry (BD, FACSCanto II, USA).

### Ovalbumin dequenching assay

The proteolytic degradation of lysosomes was assessed with DQ-ovalbumin (Invitrogen) according to the manufacturer’s instructions. DQ-ovalbumin can form green fluorescent puncta once degraded by lysosomes. In brief, after seeding in sterile culture dishes, each group of treated podocytes was incubated with 4 µg/mL DQ-ovalbumin at 37 °C for 2 h. The podocytes were fixed with 4% paraformaldehyde for 10 min and visualized using a TCS SP5 II confocal microscope. The mean fluorescent intensity of the green-fluorescent DQ-ovalbumin puncta in individual podocytes was calculated and presented as a histogram. For flow cytometric detection, after incubation with 2 µg/mL DQ-ovalbumin for 2 h, the podocytes were trypsinized and re-suspended in PBS. The fluorescent intensity value of DQ-ovalbumin was quantified by flow cytometry.

To determine whether the dequenched DQ-ovalbumin vesicles were co-localized with lysosomes, after DQ-ovalbumin incubation, the cells were fixed with 4% paraformaldehyde at room temperature and permeabilized with ice-cold methanol at −20 °C for 10 min, respectively. Following washes in PBS, the cells were labeled with mouse anti-LAMP1 (Abcam) and visualized by incubation with the secondary antibody. DAPI was used to stain the nuclei. Images were obtained by a TCS SP5 II confocal microscope.

### Plasmid transfection

Podocytes were transfected with the tfLC3 or tfGal3 plasmid (Addgene, Cambridge, MA, USA) using Lipofectamine 3000 Transfection Kit (Invitrogen) according to the manufacturer’s instructions. The podocytes transfected with the tfLC3 or tfGal3 plasmid were then treated with HIS (10%), ZAS (10%), CQ (10 µM), or rapamycin (10 µM) for an additional 4 to 36 h to assess autophagosome and autolysosome formation or lysosomal rupture as described previously^34, 39^.

### FITC annexin V/PI assays for podocyte apoptosis

Podocyte apoptosis was also determined by the Annexin V-FITC Apoptosis Detection Kit (Dojindo, Kumamoto, Japan) according to the manufacturer’s instructions. In brief, podocytes were washed with PBS, trypsinized, and re-suspended in binding solution, and then 5 μL of AnnexinV-FITC and 5 μL PI were added to 200 μL of the podocyte-containing solution and incubated at room temperature in the dark for 15 min. Apoptosis rates, representing both early apoptotic (Annexin V+/PI−) and late apoptotic (Annexin V+/PI+) podocytes, were detected with flow cytometry.

### Western blot analysis

Western blot analysis was performed as described previously^25^. The primary antibodies against LC3B, p62, cleaved caspase-3, *BECN1*, and tubulin (Abcam), and horseradish peroxidase-conjugated secondary antibodies (Beyotime Institute of Biotechnology, Jiangsu, China) were used.

### Statistical analysis

All of the statistical tests were performed using SPSS 16.0. Data are expressed as the means ± standard deviation (SD), or as counts or percentages as relevant. Two-group comparisons were performed using an independent-sample t-test unless otherwise indicated. Multiple-group comparisons were performed using analysis of variance followed by Bonferroni or Dunnett post-hoc tests. P < 0.05 was considered to be statistically significant.
